# The secret lives of single cells

**DOI:** 10.1111/1751-7915.13806

**Published:** 2021-03-26

**Authors:** Thomas K. Wood

**Affiliations:** ^1^ Department of Chemical Engineering Pennsylvania State University University Park Pennsylvania 16802‐4400 USA

## Abstract

Looking back fondly on the first 15 years of *Microbial Biotechnology*, a trend is emerging that biotechnology is moving from studies that focus on whole‐cell populations, where heterogeneity exists even during robust growth, to those with an emphasis on single cells. This instils optimism that insights will be made into myriad aspects of bacterial growth in communities.

*Microbial Biotechnology* is special to me as my group published the first two research papers in the new journal: ‘*Pseudomonas aeruginosa* PAO1 virulence factors and poplar tree response in the rhizosphere’, vol. 1 pages: 17–29, 24 August 2007 (cited 73 times to date) (Attila *et al*., 2008) and ‘Metabolic engineering to enhance bacterial hydrogen production’, vol. 1 pages: 30‐39, 24 August 2007 (cited 153 times to date) (Maeda *et al*., 2008). In the first issue of the second year, we published another manuscript, ‘Indole and 7‐hydroxyindole diminish *Pseudomonas aeruginosa* virulence’, vol 2 pages: 75–90, 22 December 2008 (cited 188 times to date) (Lee *et al*., 2009). I am proud of these three manuscripts and congratulate the editors on the successful launch of their journal. Their vision was to create a journal where high‐quality work could receive rapid review and dissemination, and they achieved their aims.

In two of these pioneering manuscripts, we used DNA microarrays to determine the transcriptome of the whole population of *P. aeruginosa* cells responding either to poplar tree roots (Attila *et al*., 2008) or to indole (Lee *et al*., 2009). By measuring the whole‐population transcriptome, we discovered seven novel *P. aeruginosa* virulence genes this organism uses with plants and discovered indole is an inter‐species signal from *Escherichia coli* that quenches quorum signalling of non‐indole‐synthesizing *P. aeruginosa* cells without affecting their growth. This led to numerous discoveries such as indole (i) prevents the resuscitation of *P. aeruginosa* persister cells (Zhang *et al*., 2019), (ii) kills bacterial and archaeal persister cells (Hu *et al*., 2015; Kwan *et al*., 2015a; Lee *et al*., 2016; Megaw and Gilmore, 2017; Li *et al*., 2019; Song *et al*., 2019; Manoharan *et al*., 2020; Sun *et al*., 2020; Yam *et al*., 2020), (iii) helps prevent infections as an *interkingdom* signal in the gastrointestinal tract by tightening epithelial cell junctions (Bansal *et al*., 2010; Shimada *et al*., 2013), (iv) regulates ageing in mice (Powell *et al*., 2020) and (v) influences brain development via the aryl‐hydrocarbon receptor (Spichak *et al*., 2021). Therefore, these early publications that made use of whole‐population studies in *Microbial Biotechnology* had a sizeable impact.

It is fascinating now that the field is moving rapidly from studying whole‐cell populations, as we did in the early *Microbial Biotechnology* manuscripts, to determining the transcriptome of *single* bacterial cells. The logical progression was from DNA microarrays of whole‐cell populations to RNA‐seq of whole‐cell populations to RNA‐seq of single cells. For the single‐cell studies, to date, there have been four main contributions of this RNA‐seq technique in bacteria and one single‐molecule fluorescence *in situ* hybridization (FISH) contribution. The first published method (25 May 2020, PETRI‐seq) was that of Blatmann et al. (2020) which identified 200 *E. coli* transcripts per exponentially growing cell as well as identified rare prophage induction in *Staphylococcus aureus* cells. The second published method (17 August 2020, MATQ‐seq) was that of Imdahl *et al*. (2020) which quantified the impact of growth on expression of 170 *Salmonella enterica* serovar Typhimurium genes and 102 *Pseudomonas aeruginosa* genes. Next, Kuchina et al. (2020) (17 December 2020, microSPLiT) were able to detect 235 transcripts/cell for *E. coli* and 397 transcripts/cell for *B. subtilis* at different growth stages. Most recently (10 March 2021), McNulty et al. (McNulty *et al*., 2021) sequenced 15,000 cells and detected 265 transcripts/*B. subtilis* cells and 149 transcripts/*E. coli* cell. In a different approach, Dar *et al*. (2021) used par‐seqFISH to *spatially* resolve and quantify hundreds of transcripts within a single *P. aeruginosa* biofilm cell (25 February 2021). Clearly, this area is moving rapidly, but it already allows one to determine which genes are expressed in a single cell as well as to identify in which specific part of the cell that the expression occurs.

The impact of discerning the response of single cells will be huge. For example, already for persister cells, single‐cell observations have led to the discovery that the mechanism by which persister cells form as well as how they resuscitate is based on their active ribosome content. Persister cells are a subpopulation of cells which arise due to stress (e.g. antibiotic, nutritive, oxidative) and weather the stress by becoming dormant (Wood and Song, 2020). Specifically, single‐cell studies were used to discover that persister cells become dormant by mothballing their protein synthesis machinery by making 100S ribosome dimers based on (p)ppGpp and cAMP signalling and through the actions of the ribosome‐inactivating proteins ribosome‐associated inhibitor A (RaiA), ribosome modulation factor (RMF) and ribosome hibernation‐promoting factor (Hpf) (Kim *et al*., 2018; Song and Wood, 2020; Wood and Song, 2020). Moreover, dormant persister cells resuscitate, upon the removal of the stress and the presence of nutrients, by activating the mothballed ribosomes via nutrient sensing through membrane chemotaxis proteins and sugar transport proteins, which leads to a reduction in the (p)ppGpp and cAMP signals and activation of HflX (Yamasaki *et al*., 2020). When they wake, the formerly dormant cells grow exponentially like wild‐type cells (Kim *et al*., 2018). This ribosome‐based mechanism was completely obscured by the previous whole‐cell population studies that tried to evaluate behaviour based on population lag times. Critically, since persister cells reconstitute infections, their mechanism of formation and resuscitation is important since over two million people a year die from bacterial infections currently, and this total is projected to increase to 10 million by 2050 at a cost of $100 trillion (Thappeta *et al*., 2020). Furthermore, although inhibiting cAMP and (p)ppGpp would likely have pleiotropic effects, these mechanistic insights suggest RaiA, RMF (conserved in gammaproteobacterial), Hpf (conserved in bacteria and all domains of life), and Hflx (conserved GTPase from bacteria to humans) are excellent targets to prevent persistence; i.e., if cells fail to mothball their ribosomes, they remain susceptible to antimicrobials.

In a similar manner, the single‐cell approach should lead to breakthroughs regarding our understanding how toxin/antitoxin (TA) systems function (Bruggeman, 2021, unpublished data) and how antibiotic resistance arises. TA systems are categorized into seven main types based on the function of the antitoxin (Wang *et al*., 2020), and multiple TA systems are found in almost all genomes (Yamaguchi *et al*., 2011). Although prevalent, their role in cell physiology is somewhat controversial, although TA systems have a clear role in phage inhibition (Pecota and Wood, 1996; Hazan and Engelberg‐Kulka, 2004; Fineran *et al*., 2009), plasmid stabilization (Ogura and Hiraga, 1983), mobile genetic element stabilization (Wozniak and Waldor, 2009; Soutourina, 2019), plasmid copy number control (Ni *et al*., 2021), and biofilm formation (Ren *et al*., 2004; Kim *et al*., 2009).

As a controversial and well‐studied example, the type II TA system MqsR/MqsA was first identified as active in *E. coli* biofilms (Ren *et al*., 2004) and shown, based on both deletion and overexpression studies, to protect *E. coli* from the bile acid it encounters in the gastrointestinal tract (Kwan *et al*., 2015b) as well as to take part in the general stress response by regulating the master regulator of the stress response, sigma factor RpoS (Wang *et al*., 2011). MqsR/MqsA have also has been found to have an effect in non‐*E. coli* systems including copper stress (Merfa *et al*., 2016), vesicles (Santiago *et al*., 2016), and biofilm formation (Lee *et al*., 2014) in *Xylella fastidiosa* as well as biofilm formation in *Pseudomonas fluorescens* (Wang *et al*., 2019), and persistence and biofilm formation in *Pseudomonas putida* (Sun *et al*., 2017).

However, two recent reports questioned the impact of MqsR/MqsA on cell physiology. The Van Melderen group claimed there was no induction of *mqsRA* and no phenotype upon deleting *mqsRA* during stress (Fraikin *et al*., 2019). A few months later, the Laub group invalidated the claim of no transcription response during stress by showing *mqsRA* was induced dramatically (181 fold) during amino acid stress and during oxidative stress (90 fold) (LeRoux *et al*., 2020). The Laub group also failed to find a phenotype for MqsR/MqsA during stress (LeRoux *et al*., 2020), although bile acid stress was not investigated, biofilms were not investigated, and their results are flawed in that they relied on the use of a TA system deletion strain that has many non‐related mutations (large chromosomal inversions) (Goormaghtigh *et al*., 2018). Note the use of TA system deletion strains with coding errors have led to notorious errors in the field that have led to three retractions, based on the errors we have described (Wood and Song, 2020). Critically, both of these studies that failed to find a phenotype with MqsR/MqsA used whole‐population studies and therefore probably missed MqsR toxin expression in a subpopulation of cells. MqsR is very toxic; i.e., deletion of the antitoxin gene is lethal (Baba *et al*., 2006), so it is likely only a few molecules of this powerful RNase enzyme are produced and are produced in a subpopulation of cells (Bruggeman, 2021, unpublished data).

Similarly, breakthroughs in understanding how antibiotic resistance arises will likely be achieved by studying single cells. Currently, it is clear antibiotic resistance arises from a series of mutations in non‐dormant bacteria that gradually change lag phase (Santi *et al*., 2021) and metabolism (Lopatkin *et al*., 2021). However, so far, these studies are limited to studying whole‐cell populations rather than single cells. Just as whole‐population studies were unable to discern the mechanism of persister formation and resuscitation (e.g. by focussing on growth lags of large populations of cells), single‐cell studies should enable the mechanism of antibiotic resistance to be determined more robustly by following changes in a single cell.

Therefore, one can be sanguine about the mechanisms that single‐cell studies will provide in the next 10 years. Compared to coarse microarray studies on whole populations of cells, such as one for *E. coli* biofilm cells developed on glass wool (Ren *et al*., 2004), which led to the discovery of the TA systems Hha/TomB (Marimon *et al*., 2016) and MqsR/MqsA (Brown *et al*., 2009; Wang *et al*., 2011; Wang *et al*., 2013), single‐cell sequencing and other single‐cell techniques (e.g. proteomics, metabolomics, phenotype mapping, microscopy) are expected to provide myriad insights into the secret lives of single cells, including how biofilms form and function, how persister cells arise in stressed clonal populations, how TA systems impact cell physiology, how antibiotic resistance occurs, and how various protection systems are invoked and interact for lytic and temperate phages.

## Funding information

No funding information provided.

## Conflict of interest

None declared.
